# Supplementary material for: Identification and functional analysis of non-coding regulatory small RNA FenSr3 in Bacillus amyloliquefaciens LPB-18
Source: PeerJ. 2023 May 15;11:e15236. doi: 10.7717/peerj.15236 (PMC10194069; doi:10.7717/peerj.15236)
Supplement: Supplemental Information 4 [file peerj-11-15236-s004.zip › KO/CK-vs-T1_map/map04919.html]

KEGG PATHWAY: Thyroid hormone signaling pathway - Reference pathway


|  |  |
| --- | --- |
| **Thyroid hormone signaling pathway - Reference pathway** |  |

[
Pathway menu
| Organism menu
| Pathway entry
| Show description
| User data mapping
]

The thyroid hormones (THs) are important regulators of growth, development and metabolism. The action of TH is mainly mediated by T3 (3,5,3'-triiodo-L-thyronine). Thyroid hormones, L-thyroxine (T4) and T3 enter the cell through transporter proteins. Although the major form of TH in the blood is T4, it is converted to the more active hormone T3 within cells. T3 binds to nuclear thyroid hormone receptors (TRs), which functions as a ligand-dependent transcription factor and controls the expression of target genes (genomic action). Nongenomic mechanisms of action is initiated at the integrin receptor. The plasma membrane alpha(v)beta(3)-integrin has distinct binding sites for T3 and T4. One binding site binds only T3 and activates the phosphatidylinositol 3-kinase (PI3K) pathway. The other binding site binds both T3 and T4 and activates the ERK1/2 MAP kinase pathway.

|  |  |  |
| --- | --- | --- |
| Reference pathway | 184% 150% 122% 100% 82% 67% 55% | 图片下载 |
